# Cerebral Microbleeds, Cerebrospinal Fluid, and Neuroimaging Markers in Clinical Subtypes of Alzheimer's Disease

**DOI:** 10.3389/fneur.2021.543866

**Published:** 2021-04-06

**Authors:** Masaki Ikeda, Sayaka Kodaira, Hiroo Kasahara, Eriko Takai, Kazuaki Nagashima, Yukio Fujita, Kouki Makioka, Kimitoshi Hirayanagi, Natsumi Furuta, Minori Furuta, Etsuko Sanada, Ayumi Kobayashi, Yasuo Harigaya, Shun Nagamine, Noriaki Hattori, Yuichi Tashiro, Kazuhiro Kishi, Hirotaka Shimada, Takayuki Suto, Hisashi Tanaka, Yasujiro Sakai, Tsuneo Yamazaki, Yukiko Tanaka, Yuko Aihara, Masakuni Amari, Haruyasu Yamaguchi, Koichi Okamoto, Masamitsu Takatama, Kenji Ishii, Tetsuya Higuchi, Yoshito Tsushima, Yoshio Ikeda

**Affiliations:** ^1^Department of Neurology, Gunma University Graduate School of Medicine, Maebashi, Japan; ^2^Department of Neurology, Geriatrics Research Institute and Hospital, Maebashi, Japan; ^3^Division of Common Education (Neurology), Faculty of Health and Medical Care, Saitama Medical University, Hidaka, Japan; ^4^Department of Diagnostic Radiology and Nuclear Medicine, Gunma University Graduate School of Medicine, Maebashi, Japan; ^5^Department of Neurology, Maebashi Red Cross Hospital, Maebashi, Japan; ^6^Department of Neuropsychiatry, Jomo Hospital, Maebashi, Japan; ^7^Department of Neurology, Mito Medical Center, Mito, Japan; ^8^Department of Radiology, Gunma University Hospital, Maebashi, Japan; ^9^Department of Neuropsychiatry, Tanaka Hospital, Yoshioka, Japan; ^10^Department of Occupational Therapy, Gunma University Graduate School of Health Sciences, Maebashi, Japan; ^11^Department of Geriatric Medicine, Uchida Hospital, Numata, Japan; ^12^Department of Neurology, Shinozuka Hospital, Fujioka, Japan; ^13^Tokyo Center for Dementia Research and Practices, Tokyo, Japan; ^14^Team for Neuroimaging, Tokyo Metropolitan Institute of Gerontology, Tokyo, Japan

**Keywords:** Alzheimer's disease, cerebral microbleeds, posterior cortical atrophy, CSF biomarkers, ^99*m*^Tc ECD-SPECT, ^11^C PiB-PET, logopenic variant of primary progressive aphasia

## Abstract

Lobar cerebral microbleeds (CMBs) in Alzheimer's disease (AD) are associated with cerebral amyloid angiopathy (CAA) due to vascular amyloid beta (Aβ) deposits. However, the relationship between lobar CMBs and clinical subtypes of AD remains unknown. Here, we enrolled patients with early- and late-onset amnestic dominant AD, logopenic variant of primary progressive aphasia (lvPPA) and posterior cortical atrophy (PCA) who were compatible with the AD criteria. We then examined the levels of cerebrospinal fluid (CSF) biomarkers [Aβ1-42, Aβ1-40, Aβ1-38, phosphorylated tau 181 (P-Tau), total tau (T-Tau), neurofilament light chain (NFL), and chitinase 3-like 1 protein (YKL-40)], analyzed the number and localization of CMBs, and measured the cerebral blood flow (CBF) volume by ^99m^Tc-ethyl cysteinate dimer single photon emission computerized tomography (^99m^Tc ECD-SPECT), as well as the mean cortical standard uptake value ratio by ^11^C-labeled Pittsburgh Compound B-positron emission tomography (^11^C PiB-PET). Lobar CMBs in lvPPA were distributed in the temporal, frontal, and parietal lobes with the left side predominance, while the CBF volume in lvPPA significantly decreased in the left temporal area, where the number of lobar CMBs and the CBF volumes showed a significant inversely correlation. The CSF levels of NFL in lvPPA were significantly higher compared to the other AD subtypes and non-demented subjects. The numbers of lobar CMBs significantly increased the CSF levels of NFL in the total AD patients, additionally, among AD subtypes, the CSF levels of NFL in lvPPA predominantly were higher by increasing number of lobar CMBs. On the other hand, the CSF levels of Aβ1-38, Aβ1-40, Aβ1-42, P-Tau, and T-Tau were lower by increasing number of lobar CMBs in the total AD patients. These findings may suggest that aberrant brain hypoperfusion in lvPPA was derived from the brain atrophy due to neurodegeneration, and possibly may involve the aberrant microcirculation causing by lobar CMBs and cerebrovascular injuries, with the left side dominance, consequently leading to a clinical phenotype of logopenic variant.

## Introduction

Cerebral amyloid angiopathy (CAA) is caused by amyloid beta (Aβ) accumulation and is characterized by several pathological changes in the walls of small cortical and leptomeningeal capillaries, arterioles, and arteries (1–4). Lobar cerebral microbleeds (CMBs) are a neuroimaging marker of CAA, thought to reflect leakage of blood products and hemosiderin deposits from cerebral vessels damaged by Aβ deposition in Alzheimer's disease (AD) (5–7). The clinical presentations of atypical AD consist not only of amnestic symptoms, but also of language/speech disturbances and visuospatial cognitive deficits. Logopenic variant of primary progressive aphasia (lvPPA) (8–10) has been subsequently confirmed by the pathological findings of AD (11), and posterior cortical atrophy (PCA) (12, 13) has been reported to be mainly based upon AD pathology (14). The three current global criteria characterizing AD include the two atypical presentations of lvPPA and PCA with memory loss and progressive cognitive decline (15–17). With regards to lvPPA and PCA, several reports have revealed the important evidences from neuroimaging (18–20) and cerebrospinal fluid (CSF) AD biomarkers (21–24). However, investigations of the four subtypes of AD (early onset AD (EOAD), late onset AD (LOAD), lvPPA, and PCA) have been scarcely reported in relation to CSF biomarkers and neuroimaging with lobar CMBs. While the current well-established CSF biomarkers for AD diagnosis include Aβ1-42, phosphorylated tau 181 (P-Tau) and total tau (T-Tau) (15–17), non-Aβ or non-tau CSF biomarker, neurofilament light chain (NFL) and chitinase 3-like 1 protein (YKL-40) have also became a point of focus as alternative biomarkers for AD (25–27). Quite recently, CSF levels of Aβ1-38 was reported to be lower in the CAA patients than the AD patients and the control, while CSF NFL of CAA and AD patients was higher than control cases (28), although they were not atypical AD phenotypes. From these findings, we postulated that differences might exist in the CSF levels of NFL, YKL-40, and Aβ1-38, the number and distribution of cortical areas, the lateral predominance (left-right difference) of lobar CMBs localization, as well as the regional CBF (cerebral blood flow) volume and ^11^C PiB retention may be apparent between the four subtypes of AD. Accordingly, we formulated the following hypotheses: (1) typical amnestic AD and atypical AD exhibit unique clinical characteristics, CSF biomarkers, and frequency of apolipoprotein E gene (APOE) ε4 allele carriers; (2) the smaller soluble Aβ molecule, Aβ1-38, and the alternative biomarkers, NFL and YKL-40, could serve as CSF biomarkers of the AD subtypes; (3) the number of lobar CMBs at the cerebral areas, as well as the laterality predominance differs among the four AD subtypes; (4) regional CBF volume differs across the four AD subtypes and is correlated with the number of CMBs at the cerebral areas. To test these hypotheses and explore the heterogeneity of clinical AD, we sought to investigate the relationships between CMBs, CSF markers, CBF volumes, and ^11^C PiB retention among the four AD subtypes.

## Materials and Methods

### Participants

The spouse or family members of each AD patient provided written informed consent for the patient to participate in this study. The subjects who underwent lumbar punctures were recruited at the Gunma University Hospital, the Geriatrics Research Institute and Hospital, and the Maebashi Red Cross Hospital. Upon entering the study, subjects underwent a standardized clinical assessment, including medical history, physical and neurological examinations, neuropsychological examinations of Mini-Mental State Examination (MMSE) (29), Montreal Cognitive Assessment (MoCA) (30), Frontal assessment battery (FAB) (31), and brain MRI scanning. A diagnosis of AD was reached in patients with a score equal to, or below 23 points on the MMSE (32), combined with information from caregivers on the patients' daily activities. The diagnosis of AD was also based upon the diagnostic criteria of the National Institute of Neurological and Communicative Diseases and Stroke-Alzheimer's Disease and Related Disorders Association (NINCDS-ADRDA) (33), in addition to adapted NIA/AA criteria, DSM-5, and IWG-2 (15–17). Subjects were classified as non-demented (ND) if they scored more than 24 points on the MMSE, and/or if, based upon information on activities of daily living (ADL) provided by the family, they were considered to have a normal daily life that did not require any cognitive assistance. We classified AD patients into the following four clinical AD subtypes: (1) early-onset amnestic dominant deficit Alzheimer's disease (EOAD), in which the age at onset (AAO) of the memory disturbances is lower than 64 years old, (2) late-onset amnestic dominant deficit Alzheimer's disease (LOAD), in which AAO is 65 years or older, (3) logopenic variant type of primary progressive aphasia (lvPPA) (9), and (4) posterior cortical atrophy (PCA) (12, 13), in which the subjects initially suffer from visual agnosia and/or visuospatial cognitive deficits followed by memory loss, consistent with current diagnostic criteria for AD (15–17). All AD patients who participated in this study had CSF biomarkers and/or ^11^C PiB-PET findings consistent with an underlying AD pathology. Exclusion criteria included patients with dementia who were clinically diagnosed with corticobasal syndrome (CBS), progressive supranuclear palsy (PSP), dementia with Lewy bodies (DLB), frontotemporal dementia (FTD), vascular dementia (VaD), cerebral amyloid angiopathy-related inflammation (CAA-RI), or other neurodegenerative diseases characterized by dementia. No patient who participated in this study had autopsy performed.

### Assessments of Language/Speech Dysfunctions and Visual Agnosia/Visuospatial Cognitive Deficits

Speech function was assessed using the Standard Language Test of Aphasia (SLTA) (10, 34), a battery of tests originally developed to assess multi-domain language function, including “Confrontation naming,” “Word repetition,” “Sentence repetition,” “Auditory single-word comprehension,” and “Auditory complex sentence comprehension commands.” A proportion of patients were assessed using the WAB (35). Visual cognitive functions were assessed using the Benton Visual Retention Test (BVRT) and/or VPTA (36). These assessments were carried out for the differential diagnosis of visual agnosia/visuospatial cognitive deficits. We examined AD patients who primarily presented with language/speech deficits and were diagnosed with logopenic variant type of primary progressive aphasia (lvPPA) according to the Consensus Classification of the three clinical variants of PPA (9). We also examined AD patients who primarily presented with visual agnosia/visuospatial cognitive deficits dominant AD as posterior cortical atrophy (PCA) (12, 13).

### Analyses of CSF Levels of Aβ1-42, Aβ1-40, Aβ1-38, P-Tau, T-Tau, NFL, and YKL-40

Cerebrospinal fluid (CSF) was obtained by a lumbar puncture of the L3/L4 or L4/L5 intervertebral space, and the CSF samples were centrifuged for 10 min at 1,800 × g at 4°C within 3 h of collection. Samples were divided into aliquots of 0.5 mL in polypropylene tubes and stored at −80°C until analysis with ELISA kits for human CSF Aβ1-42 and Aβ1-40 (FUJIFILM Wako Pure Chemical Corporation, Osaka, Japan) (10, 37) and for CSF Aβ1-38 (IBL, Fujioka, Gunma, Japan) (10, 37). Inter-assay CVs (coefficients of variation) of the CSF Aβ1-42, Aβ1-40, and Aβ1-38 were <20%, respectively (Supplementary Table 1). All samples were measured by a single operator using the same reagents. Measurement of phosphorylated Tau (P-Tau) in CSF was performed using a sandwich ELISA INNOTEST® PHOSPHO-TAU (181P) (FUJIREBIO, Ghent, Belgium) as previously described elsewhere (10, 37). Human total tau (T-tau) was measured using a sandwich ELISA INNOTEST® T-Tau-Ag (FUJIREBIO, Ghent, Belgium) (38). Measurement of NFL (neurofilament light chain), a CSF marker of neurodegeneration and large fiber axonal degeneration, was performed using the sandwich ELISA NF-light® (IBL International, Hamburg, Germany) (39, 40). CSF levels of YKL-40 (chitinase 3-like 1 protein), a CSF marker of glial neuroinflammation, was measured by the MicroVue™ YKL-40 EIA kits (Quidel, San Diego, CA, USA) (40–42). Inter-assay CVs of P-Tau, T-Tau, NFL, and YKL-40 were <20%, respectively (Supplementary Table 1). All samples were measured by a single operator using the same reagents.

### Analyses of APOE Allele

After obtaining informed consent for genetic testing of the apolipoprotein E gene (APOE) allele, we purified genomic DNA from lymphocytes in the peripheral blood of affected subjects. For the analysis of APOE allele polymorphism, purified genomic DNA samples were examined as previously described (10, 37).

### Analyses of Neuroimaging Markers

#### MRI

All participants underwent a brain MRI [T2WI, T1WI, FLAIR, T2^*^WI (2-dimensional gradient recalled echo)] carried out on three different MRI scanners (Siemens 3.0T, Siemens 1.5T, and General Electric 1.5T) (Supplementary Tables 2, 3). CMBs were defined as homogenous, round areas with a signal void (of a diameter smaller than 10 mm) detected by T2^*^WI (7). Lobar CMBs were defined as microbleeds restricted primarily to cortical areas of frontal, temporal, parietal, and occipital lobes bilaterally. We quantified the number of CMBs in deep white matter (DWM) and cerebellum, rated by MRI (axial T2^*^WI) according to the anatomical rating scale (MARS) (43). The number of CMBs on MRI T2^*^WI was determined independently, and a random order, by the first rater (M.I.) who was an experienced neurologist and the second rater (H.K.) who was an excellent neuroradiologist, both of whom were blinded to the clinical diagnosis of the patients. In cases of disagreement, the number of CMBs were ascertained by consensus. The number of CMBs were used to estimated inter-rater reliability by weighted kappa coefficient (0.827) carried out by statistical analyses in SPSS 24.0 (SPSS Inc. Chicago, IL, USA). All neuro-radiological analyses were conducted by PACS Imaging Workstation (Sectra AB, Stockholm, Sweden).

#### ^99m^Tc ECD-SPECT Studies

AD patients underwent ^99m^Tc-ethyl cysteinate dimer single photon emission computerized tomography ^(99m^Tc ECD-SPECT) (FUJIFILM RI Toyama Chemical Co., Ltd., Chuo-ku, Tokyo, Japan) imaging as previously described (44). The degree of uptake of ^99m^Tc ECD-SPECT and its AD diagnostic abilities are in excellent concordance with those of ^18^F FDG-positron emission tomography (PET) (45). We assessed blood perfusion of CBF volumes in the brains of patients with the four subtypes of AD by ^99m^Tc ECD-SPECT bilaterally in five regions (frontal, temporal, parietal, occipital lobes, and cerebellum) according to previously published methodology (44, 45), however, we did not perform partial volume correction in this study. Each AD patient was placed in a supine position on the scanning bed with eyes closed during injection and during the sequent scanning period with a quiet examination dose of 600 MBq.

#### ^11^C PiB-PET Studies

^11^C PiB [2-(4-aminophenyl)-6-hydroxybenzothiazole] was synthesized for ^11^C PiB-PET (46), and ^18^F-labeled fluorodeoxyglucose was also synthesized for PET (FDG-PET) in Gunma University hospital cyclotron according to previous reported methods (10, 46–50). We used a Discovery ST Elite scanner (General Electric Medical Systems, Milwaukee, WI, USA) for all PET studies. After an intravenous injection of ^11^C-PiB (550 MBq), emission scans were acquired three-dimensionally without arterial sampling. Images were loaded on Xeleris workstation (General Electric Medical Systems, Milwaukee, WI), where ^11^C PiB-PET images were co-registered with the respective ^18^F FDG-PET images (10, 50). The ^11^C PiB-PET images were rated as “positive” by visual inspection when the uptake level in the cerebral cortex was more prominent than those in the white matter (10, 49, 50). The standardized uptake value ratio (SUVR) represents a quantitative measure of tracer uptake, which is normalized to the mean uptake in a reference region as well as published protocols (46–49) and our previous methods (50). The cerebellar cortex was selected as a reference region to evaluate the mean cortical SUVR (mcSUVR) of ^11^C PiB-PET as ^11^C PiB uptake in the cerebellar cortices does not differ between AD patients and healthy controls (46–49). Thus, since the cerebellar cortices are expected to have a lower fibrillary Aβ plaque burden than the cerebral cortices, the cerebellar cortex was used as a reference region to evaluate mcSUVR (48, 49). Regions in the frontal cortical region (FRC), parietal cortical region (PAR), anterior cingulate region (ANC), posterior cingulate region (PCG), lateral temporal lobe cortical region (LTC), medial temporal lobe cortical region (MTC), and occipital cortical region (OCC) were selected to calculate the mcSUVR of the respective areas (46–50). Circular standard regions of interest (ROI) of 1 cm in diameter were placed on each cortical region of each ^11^C PiB-PET images onto each cortical region of the ^11^C PiB-PET image using the co-registered FDG-PET image (50). However, we did not perform partial volume correction. A standardized single ROI was placed over three regions of the FRC, three of the PAR, one of the ANC, one of the PCG, three of the LTC, one of the MTC, and one of the OCC in the ipsilateral side (50). The levels of regional ^11^C PiB accumulation were summarized and the mcSUVR was calculated. Additionally, the mcSUVR at a total of 26 areas was used to calculate a global SUVR in each subject (50). Mean cortical SUVR values were calculated in 31 participants (lvPPA: 4, PCA: 4, EOAD: 10, LOAD: 13) who underwent ^11^C PiB-PET scans, in all 14 areas as described above.

### Statistical Analyses

Comparison analyses for demographic data (AAO, duration of illness, MMSE, MoCA, FAB, education years, hypertension, diabetes, hypercholesterolemia, and APOE ε4 allele carrier) were performed between the two clinical AD subgroups (the typical AD group and the atypical AD group) using either Mann-Whitney-tests for continuous variables, or a chi-squared or Fisher's-test for categorial variables. The chi-squared-test applied for categorical variables, was also used to evaluate the association between the number of microbleeds between the two AD subtypes. Statistical comparisons of CSF Aβ1-38, Aβ1-40, Aβ1-42, P-Tau, T-Tau, NFL, and YKL-40 across the two AD subgroups and the ND group were performed using a one-way analysis of variance (ANOVA) (*p* < 0.05), Tuckey's-test was used for *post-hoc* comparison. If the non-normal distribution was identified for non-parametrical comparison using Mann-Whitney tests (defined as *p* < 0.05), Dunn's test-was used for *post-hoc* comparison.

A Kruskal-Wallis-test, as well as one-way ANOVA and Dunn's-test, used for *post-hoc* comparison and correction for multiple comparisons, were applied to the following analyses: statistical comparisons of the CSF biomarkers across the four AD subgroups, comparison of CBF volumes by ^99m^ECD-SPECT among the frontal, temporal, parietal, and occipital lobe cortices and cerebellum, and comparisons of ^11^C PiB retention among the frontal, temporal, parietal, and occipital lobe cortices. The correlation analysis among levels of the CBF volumes and the number of lobar CMBs was performed using Spearman's rank correlation tests at the four areas (frontal, temporal, parietal, and occipital) in the four AD subtypes. Correlation analysis for the levels of CSF markers and number of lobar CMBs was performed using Spearman's rank correlation tests in all AD patients.

Data were reported as mean ± SD (standard deviation). All statistical analyses were performed using SPSS software package (version 24: SPSS Inc., Chicago, IL, USA) applying a significance level of p < 0.05, and graphs were drafted using GraphPad Prism 7 (GraphPad Software, La Jolla, CA, USA).

### Standard Protocol Approvals and Patient Consent

This study complied with the Declaration of Helsinki and was approved by the Gunma University Ethical Review Board for Medical Research Involving Human Subjects of Gunma University (Maebashi, Gunma, Japan), the Geriatrics Research Institute and Hospital (Maebashi, Gunma, Japan), and Maebashi Red Cross Hospital (Maebashi, Gunma, Japan). The spouse or family members of each AD patient provided written informed consent for the patient to participate in the study.

## Results

### Demographics and CSF Biomarkers in Typical AD and Atypical AD

A total of 117 AD patients were enrolled, then divided into the 85 typical amnestic AD patients (39 EOAD and 46 LOAD) and the 32 atypical AD patients (20 lvPPA and 12 PCA). Clinical information of the AD patients and the 40 non-demented (ND) subjects, and CSF biomarkers of them (Aβ1-42, Aβ1-40, Aβ1-38, P-Tau, T-tau, NFL, and YKL-40) were also investigated (Table 1). CSF levels of Aβ1-42 were significantly lower in the typical AD (*n* = 85) and atypical AD (*n* = 32) groups compared to the ND group (*n* = 40) (*p* < 0.0001, respectively; Table 1). Meanwhile, CSF levels of P-Tau, T-Tau, NFL, and YKL-40 were significantly higher in both AD group compared to the ND group (*p* < 0.0001, respectively; Table 1). Additionally, the CSF levels of Aβ1-38 were significantly lower in the atypical AD compared to the typical AD group (*p* = 0.002), although the results of other CSF biomarkers and the prevalence of APOE ε4 allele did not differ significantly between the typical AD and atypical AD groups (Table 1).

**Table 1:** Demographics and CSF biomarkers in typical/atypical AD patients.

	**Typical AD**	**Atypical AD**	**ND**	**Typical AD vs. atypical AD(*p*-value)**
No.	85	32	40	
Male %	42.05	53.13	50.00	0.307 (χ^2^)
Age at onset (years)	65.99 ± 11.08	65.09 ± 8.35	NA	0.379
Duration of illness (years)	3.24 ± 2.07	3.50 ± 2.34	NA	0.755
Age at lumber puncture	69.75 ± 8.43	68.59 ± 7.61	NA	0.429
Education (years)	12.02 ± 1.72	12.09 ± 1.11	NA	0.727
Hypertension (%)	18 (21.18)	5 (18.52)	NA	0.680 (χ^2^)
Diabetes (%)	12 (14.12)	3 (9.38)	NA	0.709 (χ^2^)
Hypercholesterolemia (%)	15 (17.65)	5 (18.5)	NA	0.987 (χ^2^)
MMSE (/30)	19.34 ± 4.82^*^	17.81 ± 4.32^*^	29.15 ± 1.05	0.075
MoCA (/30)	14.65 ± 4.85	12.53 ± 4.05	NA	0.017
FAB (/18)	8.74 ± 3.23	7.38 ± 3.49	NA	0.081
APOE ε4 (%)	57.65	46.88	NA	0.214 (χ^2^)
CSF Aβ1-42 (pg/ml)	182.81± 58.07^*^	176.7 ± 29.66^*^	425.06 ± 126.31	0.752
CSF Aβ1-40 (pg/ml)	4,503 ± 1,745	4,193 ± 1,085	7062.96 ± 4163.42	0.616
CSF Aβ1-38 (pg/ml)	3,255 ± 1,181	2,183 ± 629.5	2533.64 ± 1253.03	0.002
CSF P-Tau (pg/ml)	83.07 ± 31.63^*^	78.27 ± 34.19^*^	36.37 ± 14.17	0.409
CSF T-Tau (pg/ml)	593.95 ± 175.75^*^	578.21 ± 232.20^*^	151.50 ± 72.27	0.717
CSF NFL (pg/ml)	1,906 ± 1,140^*^	2,468 ± 1,681^*^	579.89 ± 322.96	0.306
CSF YKL-40 (ng/ml)	120.41 ± 48.14^*^	124.16 ± 49.93^*^	60.53 ± 22.25	0.681

*Differences between groups were analyzed using a chi-squared-test for the categories of sex, hypertension, diabetes, and hypercholesterolemia, and a Kruskal-Wallis-test, and variance was analyzed using post-hoc Dunn-tests (for age at onset, duration of illness, years of education, and total scores of MMSE, MoCA, and FAB, respectively). Data are presented as mean ± standard deviation. NA, not applicable; ND, non-demented subject*.

* *p < 0.0001 comparing the ND and the typical and atypical AD groups combined*.

### Demographics of the Four Subtypes of AD Patients

We then classified the AD patients into EOAD (*n* = 39), LOAD (*n* = 46), lvPPA (*n* = 20), and PCA (*n* = 12) according to the AD criteria (15–17). The AAO of PCA and EOAD were significantly lower than that of LOAD (*p* < 0.0001, *p* < 0.0001, respectively), and the AAO of PCA was significantly lower than that of lvPPA (*p* = 0.046), however, higher than that of EOAD (*p* = 0.0002). The total MoCA scores in lvPPA were significantly lower than those in LOAD (*p* = 0.015), while the total FAB scores in lvPPA were significantly lower than those in EOAD (*p* = 0.045; Table 2). In the neuropsychological examinations, the scores of auditory sentence comprehension of lvPPA were significantly lower than those of PCA (*p* = 0.015), EOAD (*p* = 0.042), and LOAD (*p* = 0.0005). The “intersecting pentagon” scores of PCA were significantly lower than those of EOAD (*p* = 0.021) and LOAD (*p* = 0.0009). The “Clock drawing test” scores of PCA were significantly lower than those of LOAD (*p* = 0.028). Additionally, the SLTA was performed as previously described for speech/language function (10), which revealed that the lvPPA patients presented with the hesitant speech and word-finding pauses due to impaired single word retrieval and difficulty with sentence repetition (data not shown), which agreed with the criteria described for lvPPA (8, 9).

**Table 2:** Demographics of the four subtypes of AD patients.

	**lvPPA**	**PCA**	**EOAD**	**LOAD**	**Comparing AD subtypes ANOVA (*p*-value)**	***Post*-*hoc* differences (*p*-value)**
No.	20	12	39	46		
Male %	55.00	50.00	38.46	45.65	0.05 < *p* (χ^2^)	
Age at onset (years)	68.70 ± 6.95	60.00 ± 8.34	57.31 ± 10.41	73.35 ± 4.13	<0.0001	PCA < lvPPA (0.046)
						EOAD < PCA (0.0002)
						PCA < LOAD (<0.0001)
						EOAD < LOAD (<0.0001)
Duration of illness (years)	3.65 ± 2.58	3.25 ± 1.96	3.92 ± 2.53	2.98 ± 2.04	0.315	
Education (years)	11.58 ± 1.04	12.75 ± 2.34	12.18 ± 1.32	12.02 ± 0.91	0.085	
MMSE (/30)	17.10 ± 4.12	19.00 ± 4.57	18.67 ± 4.89	19.91 ± 4.73	0.101	
MoCA (/30)	11.35 ± 3.88	13.83 ± 4.15	14.21 ± 5.10	15.02 ± 4.65	0.025	lvPPA < LOAD (0.015)
FAB (/18)	6.35 ± 3.15	9.08 ± 3.50	8.79 ± 3.69	8.70 ± 2.82	0.039	lvPPA < EOAD (0.045)
Hypertension (%)	3 (15)	3 (25)	7 (17.95)	10 (21.74)	0.05 < *p* (χ^2^)	
Diabetes mellitus (%)	2 (10)	1 (8.33)	6 (15.38)	11 (23.91)	0.05 < *p* (χ^2^)	
Hypercholestrolemia (%)	4 (20)	2 (16.67)	6 (15.38)	12 (26.09)	0.05 < *p* (χ^2^)	
**Language**						
Confrontation naming (0–2)	1.45 ± 0.83	1.67 ± 0.49	1.85 ± 0.43	1.76 ± 0.48	0.149	
Single word repetition (0–3)	2.20 ± 1.24	2.75 ± 0.45	2.56 ± 0.72	2.61 ± 0.68	0.719	
Single word recall (0–3)	0.60 ± 0.75	0.75 ± 0.86	0.82 ± 0.88	0.89 ± 1.08	0.849	
Sentence repetition (0–1)	0.40 ± 0.50	0.75 ± 0.45	0.69 ± 0.47	0.65 ± 0.48	0.112	
Auditory sentence comprehension (0–3)	1.40 ± 1.05	2.58 ± 0.67	2.15 ± 1.09	2.48 ± 0.89	0.0007	lvPPA < PCA (0.015)
						lvPPA < EOAD (0.042)
						lvPPA < LOAD (0.0005)
**Calculation**						
Serial seven (0–5)	1.25 ± 1.07	1.33 ± 0.98	1.69 ± 1.52	1.91 ± 1.54	0.468	
Digit span backward (0–2)	0.60 ± 0.74	0.33 ± 0.49	0.54 ± 0.55	0.74 ± 0.61	0.167	
**Visuospatial functioning**						
Intersecting pentagons (0-1)	0.50 ± 0.51	0.08 ± 0.29	0.56 ± 0.50	0.69 ± 0.46	0.0022	PCA < EOAD (0.021) PCA < LOAD (0.0009)
Clock drawing test (0-3)	0.95 ± 0.99	0.42 ± 0.51	1.18 ± 0.91	1.26 ± 0.97	0.0284	PCA < LOAD (0.028)

*Differences between groups were analyzed using a chi-squared-test for the categories of sex, hypertension, diabetes, and hypercholesterolemia, and a Kruskal-Wallis-test, and variance was analyzed using post-hoc Dunn-tests (for age at onset, duration of illness, years of education, total scores of MMSE, MoCA, FAB, respectively, and the sub-scores of Language, Calculation, Visuospatial functioning, and the Clock drawing test)*.

### Prevalence and Localization of CMBs in the Four Clinical AD Subtypes

**Figure 1:**
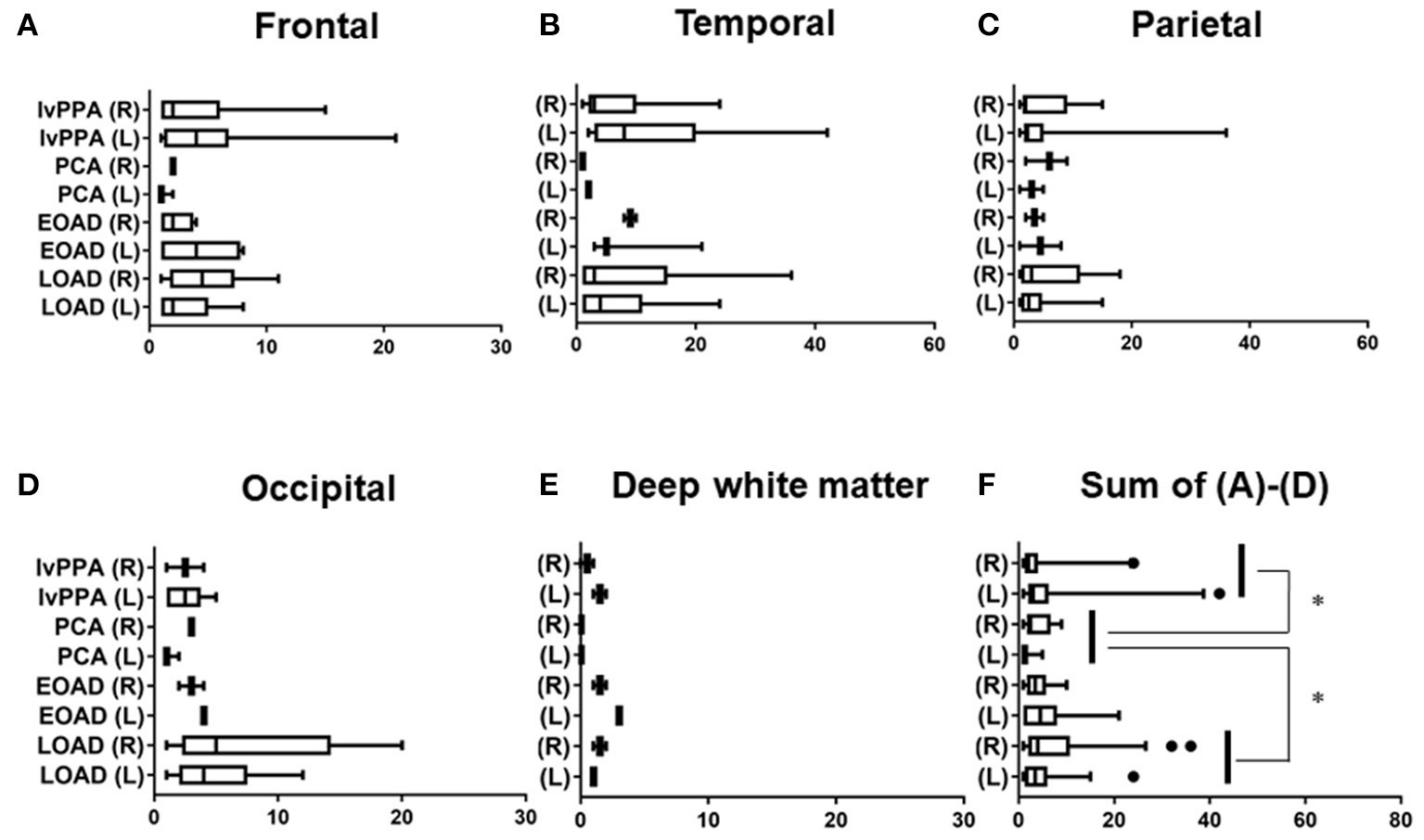
Regional comparison of the number of CMBs in the four subtypes of AD. Number of lobar CMBs in frontal **(A)**, temporal **(B)**, parietal **(C)**, occipital **(D)** lobes, and deep white matter (DWM) **(E)** of lvPPA, PCA, EOAD, and LOAD. Total number of lobar CMBs in frontal, temporal, parietal and occipital lobe cortices **(F)**. *p < 0.05.

The prevalence of lobar CMBs (1≦) in lvPPA (50.00%) was higher than those in the other three AD subtypes (EOAD: 23.08%, PCA: 25.00%, LOAD: 36.96%). Among the four AD subtypes, the prevalence of lobar CMBs in lvPPA was significantly higher than those in both PCA (*p* = 0.036), as well as higher, though not significantly, than EOAD (*p* = 0.082) and LOAD (*p* = 0.322; Table 3). Comparison of the mean number of lobar CMBs per an AD patient with lobar CMBs was not significantly different across the four AD subtypes in the bilateral sides (*p* = 0.312), the right side (*p* = 0.715) and the left side (*p* = 0.259; Table 3). The number of lobar CMBs in lvPPA tended to be higher in the left side than the right side, although comparison of the mean number of lobar CMBs between the right and left sides was not significant among the four AD subtypes (*p* = 0.161; Table 3). Moreover, while the number of lobar CMBs in the frontal, temporal, and parietal lobes in lvPPA and LOAD tended to be higher than in PCA (Figures 1A–C), the number of lobar CMBs in the occipital area in LOAD tended to be higher than in the other subtypes of AD (Figure 1D). Additionally, the total number of lobar CMBs in the frontal, temporal, parietal, and occipital lobes in lvPPA and LOAD was significantly higher than in PCA (*p* = 0.0332, *p* = 0.0136, respectively, Figure 1F). There was no significant difference in the number of CMBs in DWM among the four AD subtypes, although the number of CMBs on each side of the DWM was ≦ 3 in each AD patient (Figure 1E). Among the four AD subtypes with lobar CMBs (lvPPA: *n* = 10, PCA: *n* = 3, EOAD: *n* = 9, LOAD: *n* = 17), lobar CMBs in lvPPA tended to be predominantly localized on the left side, rather than those on the right side of frontal, temporal, parietal, and occipital lobes (left side percentage of frontal (68.18%), temporal (65.91%), parietal (67.74%), occipital (70.00%) lobes and total areas (67.52%) shown in Figures 1A–D,F). The total number of CMBs in frontal, temporal, parietal, and occipital lobe cortices in lvPPA tended to be higher in the left side compared to the right, although not significant (*p* = 0.1833; Figure 1F), while there was no difference in the laterality predominance in the other subtypes of AD.

**Table 3:** Prevalence and mean number of lobar CMBs in the four subtypes of AD patients.

	**lvPPA**	**PCA**	**EOAD**	**LOAD**	**Comparing AD subtypes ANOVA (*p-*value)**	***Post*-*hoc* differences (*p-*value)**
No.	20	12	39	46		
Lobar CMBs (1≦) (%)	10 (50.00)	4 (25.00)	9 (23.08)	17 (36.96)	0.024	EOAD < lvPPA (0.082)
						PCA < lvPPA (0.036)
						LOAD < lvPPA (0.322)
Mean Lobar CMBs No. (Bilateral) (per a patient with lobar CMBs)	11.60 ± 9.87	3.33 ± 3.22	8.39 ± 14.69	10.85 ± 10.30	0.312	
Mean lobar CMBs No. (Right) (per a patient with lobar CMBs)	4.75 ± 3.69	2.00 ± 3.64	5.20 ± 8.23	6.15 ± 6.36	0.715	
Mean lobar CMBs No. (Left) (per a patient with lobar CMBs)	7.80 ± 6.13	1.33 ± 0.33	4.50 ± 8.14	5.31 ± 3.68	0.259	
Comparing mean lobar CMBs No. (Right vs. Left) Kruskal-Wallis-test	0.161	0.600	0.987	0.602		

*Data are presented as mean ± standard deviation. Differences between groups were analyzed using a chi-squared test, and a Kruskal-Wallis-test, and variance was analyzed using post-hoc Dunn-tests for mean lobar CMBs numbers (No.), respectively*.

### Diagnostic Values of CSF Biomarkers in the AD Patients

We did not have data information of CSF from AD patients based upon with post-mortem verification of AD diagnosis. We calculated diagnostic values of the CSF biomarkers of Aβ1-42 (cut off <308.7), P-Tau (cut off > 47.43), T-Tau (cut off > 270.2), NFL (cut off > 858.8), and YKL-40 (cut off > 78.59) by the data from the typical AD (EOAD and LOAD) patients (*n* = 72) and non-dementia (ND) subjects (*n* = 33). These data were shown in Table 4.

**Table 4:** Diagnostic values of CSF biomarkers in the typical AD patients vs. ND.

**Typical AD vs. ND**	**Cut-point**	**95% CI**	***p***	**Sensitivity (%)**	**Specificity (%)**
Aβ1-42	<308.7	0.8883–0.9961	<0.0001	96.77	90.91
P-Tau	>47.43	0.8411–0.9764	<0.0001	92.86	81.58
T-tau	>270.2	0.8992–0.9965	<0.0001	97.10	94.74
NFL	>858.8	0.8203–0.9674	<0.0001	91.30	85.71
YKL-40	>78.59	0.7381–0.9303	<0.0001	85.25	75.00

*AD, Alzheimer's disease; ND, non-dementia subjects; Aβ, Amyloid β; P-Tau, phosphorylated tau; T-tau, total tau; NFL, neurofilament light chain; YKL-40, chitinase 3-like 1 protein*.

### Results of CSF Biomarkers, APOE ε4 Alleles, and Global ^11^C PiB-PET Retention in the Four AD Subtypes

CSF biomarkers were analyzed in 18 patients with lvPPA, 12 with PCA, 34 with EOAD, and 38 with LOAD, in addition to the 33 ND subjects, by reference to the diagnostic values of CSF biomarkers CSF biomarkers in the typical AD patients vs. ND (Table 4). The CSF levels of Aβ1-42 (pg/ml) in the four AD subtypes were all significantly lower than those in ND (one-way ANOVA *post-hoc* Dunn's-test, *p* < 0.0001, respectively for all four AD subtypes), however, no significant differences were observed across the four AD subtypes (Kruskal-Willis-test: *p* = 0.983). The Aβ1-40 (pg/ml) CSF levels did not differ significantly from those in ND (one-way ANOVA *post-hoc* Dunn's-test, 0.05 < *p*), and no significant differences were found across the four AD subtypes (Kruskal-Willis-test: *p* = 0.379). The Aβ1-38 (pg/ml) CSF levels of the AD subtypes were not significantly different from those in ND (Kruskal-Willis-test: *p* = 0.035; *post-hoc* difference, 0.05 < *p*), nor were the CSF Aβ1-38 levels significantly different across the four AD subtypes (0.05 < *p*; Table 5).

**Table 5:** CSF biomarkers, APOE ε4 alleles and global ^11^C PiB-PET retention in the four subtypes of AD patients.

**CSF markers**	**lvPPA**	**PCA**	**EOAD**	**LOAD**	**ND**	**Comparing AD subtypes (*p-*value)**	***Post*-*hoc* differences (*p-*value)**
No.	18	12	34	38	33		
Aβ1-42	174.67 ± 29.72^a^	181.074 ± 49.88^a^	181.18 ± 58.39^a^	184.871 ± 58.68^a^	441.1 ± 90.13	0.983	
Aβ1-40	4415.82 ± 1073.98	4489.73 ± 1887.83	4202.36 ± 1585.82	4735.38 ± 1844.25	6,196 ± 1,325	0.379	
Aβ1-38	2224.77 ± 651.98	2036.91 ± 645.98	3053.18 ± 990.84	3390.02 ± 1287	2,640 ± 1,032	0.035	no significant difference
P-Tau	84.68 ± 37.05^a^	58.71 ± 28.71^c^	87.24 ± 33.45^a^	79.55 ± 30.01^a^	34.86 ± 12.84	0.389	
T-Tau	547.74 ± 234.98^a^	568.66 ± 251.81^a^	596.29 ±1 96.32^a^	592.04 ± 197.93^a^	157.89 ± 68.25	0.756	
NFL	2731.43 ± 1227.88^a^	1101.18 ± 269.37^c^	1781.57 ± 1260.64^a^	2041.86 ± 994.38^a^	541.1 ± 273.5	0.002	PCA < lvPPA (0.003)
							EOAD < lvPPA (0.014)
							LOAD < lvPPA(0.045)
YKL-40	135.54 ± 54.56^a^	96.49 ± 36.12^c^	107.96 ± 42.83^b^	130.97 ± 50.54^a^	60.53 ± 22.25	0.148	
APOE ε4 carrier (%)	10/18 (55.56)	5/12 (41.67)	22/34 (64.71)	20/38 (52.63)	–	0.646 (χ^2^)	
Global PiB retention	1.94 ± 0.48	1.99 ± 0.49	1.82 ± 0.41	1.93 ± 0.49	–	0.072	

*A Kruskal-Wallis test, one-way ANOVA multiple comparison analysis and post-hoc Dunn correction yielded the following results: CSF levels of Aβ1-42 were lower, and CSF levels of P-Tau, T-Tau, NFL, and YKL-40 were higher than those of ND*:

a *p < 0.0001*,

b *p < 0.001*,

c *p < 0.05*.

*Among the four subtypes of AD, CSF levels of NFL in lvPPA were significantly higher than those in PCA (p = 0.003), EOAD (p = 0.014), and LOAD (p = 0.045)*.

A total of 135 subjects were analyzed for the CSF levels of P-Tau, T-Tau, NFL, and YKL-40. The levels of CSF P-Tau were significantly higher in lvPPA, EOAD, LOAD, PCA compared with those in ND (ANOVA *post-hoc* Dunn's-test: *p* < 0.0001, *p* < 0.0001, *p* < 0.0001, *p* = 0.0212, respectively). Meanwhile, there were no significant differences observed in P-Tau across the four AD subtypes (Kruskal-Willis-test: *p* = 0.389). The CSF levels of T-Tau (pg/ml) were higher in lvPPA, LOAD, EOAD, and PCA than ND (*p* < 0.0001, respectively). However, no significant differences were observed in T-Tau across the four AD subtypes (Kruskal-Willis-test: *p* = 0.756). The CSF levels of NFL (pg/ml) were the highest in lvPPA, followed by LOAD, EOAD and PCA, and all four AD subgroups had higher levels than ND (*p* < 0.0001, *p* < 0.0001, *p* < 0.0001, *p* = 0.036, respectively). Across all four AD subtypes, the CSF levels of NFL in lvPPA were significantly higher than those in PCA (*p* = 0.003), EOAD (*p* = 0.014) and LOAD (*p* = 0.045). The CSF levels of YKL-40 (ng/ml) in lvPPA, LOAD, EOAD, and PCA were significantly higher than those in ND (*p* < 0.0001, *p* < 0.0001, *p* < 0.001, *p* = 0.044, respectively); meanwhile no significant difference was noted among the four AD subtypes (Kruskal-Willis-test: *p* = 0.148; Table 5). The prevalence of APOE ε4 allele carrier was the highest in EOAD (64.71%, *n* = 22/34), followed by lvPPA (55.56%, *n* = 10/18), LOAD (52.63%, *n* = 20/38), and PCA (41.67%, *n* = 5/12), with no significant differences among them (Table 5). There is no significant difference in global mcSUVR across all 14 areas among the four AD subtypes (Table 5).

### Regional Comparison of CBF Volumes in the Four Clinical Subtypes of AD

**Figure 2:**
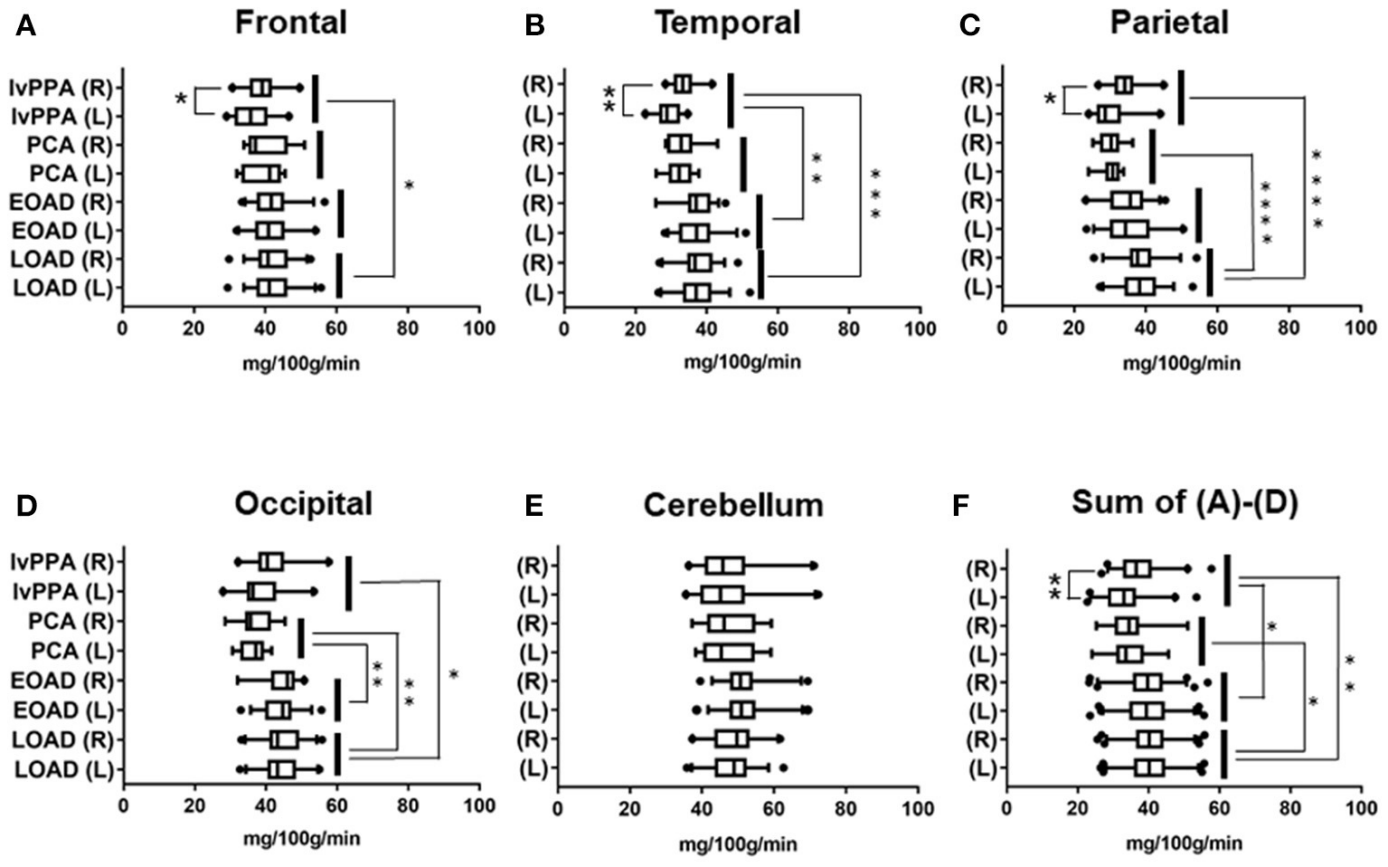
Regional comparison of CBF volumes in the four AD subtypes in ^99m^Tc ECD-SPECT. CBF volumes in frontal **(A)**, temporal **(B)**, parietal **(C)**, occipital **(D)** lobes and cerebellum **(E)** of lvPPA, PCA, EOAD, and LOAD. Total CBF volumes, namely, Sum of **(A–D)**, in frontal, temporal, parietal and occipital lobes **(F)**. *p < 0.05, **p < 0.01, ***p < 0.001, ****p < 0.0001.

The number of AD patients examined by ^99m^Tc ECD-SPECT were as follows: lvPPA: *n* = 17, PCA: *n* = 12, EOAD: *n* = 32, and LOAD: *n* = 36. In the frontal lobe, CBF volumes in lvPPA were significantly lower than those in LOAD (*p* = 0.033; Figure 2A). In the temporal lobe, the CBF volumes in lvPPA were significantly lower than those in EOAD (*p* = 0.0011) and LOAD (*p* = 0.0002; Figure 2B). In the parietal areas, the CBF volumes in PCA and lvPPA were lower than those in LOAD (*p* < 0.0001, respectively; Figure 2C). In the occipital lobe, the CBF volumes in PCA were significantly lower than those in EOAD (*p* = 0.0098) and LOAD (*p* = 0.002), while those in lvPPA were significantly lower than those in LOAD (*p* = 0.0311; Figure 2D). In the cerebellar region, there was no significant difference in the CBF volumes across all four AD subtypes (Figure 2E). Among the total CBF volumes, namely FTPO, including those of the frontal, temporal, parietal, and occipital lobes in the four AD subtypes, the mean CBF volumes in lvPPA were significantly lower than those in EOAD (*p* = 0.047) and LOAD (*p* = 0.002), while those in PCA were significantly lower than those in LOAD (*p* = 0.0132; Figure 2F). CBF volumes in lvPPA were significantly lower in the left side of the frontal, temporal, and parietal lobes than in the right side (^*^p < 0.05, ^**^p < 0.0001, respectively, shown in Figures 2A–D,F).

### Correlation Between CBF Volume and Number of Lobar CMBs in the Four AD Subtypes

Spearman rank correlation tests revealed that the levels of CBF volume were significantly inversely correlated with the number of lobar CMBs in the left temporal region in lvPPA (*r* = −0.382, *p* = 0.046); bilateral (right; left) parietal region (*r* = −0.397, *p* = 0.020; *r* = −0.345, *p* = 0.042, respectively), bilateral (right; left) occipital region (*r* = −0.353, *p* = 0.042; *r* = −0.408, *p* = 0.043, respectively), and the bilateral (right; left) temporal region (*r* = −0.345, *p* = 0.042; *r* = −0.368, *p* = 0.027) in LOAD (Table 6).

**Table 6:** Correlation between the levels of CBF volumes and the number of lobar CMBs in the four subtypes of AD patients.

**C.I. (*p-*value)**	**R/L**	**lvPPA**	**PCA**	**EOAD**	**LOAD**
No.		17	12	32	36
Frontal	R	−0.285 (0.099)	−0.412 (0.250)	−0.001 (0.498)	0.061 (0.383)
	L	−0.316 (0.076)	−0.577 (0.250)	0.119 (0.299)	−0.066 (0.375)
Temporal	R	0.106 (0.319)	0.082 (0.500)	−0.052 (0.410)	−0.345 (0.042)
	L	−0.382 (0.046)	−0.252 (0.321)	0.052 (0.410)	−0.368 (0.027)
Parietal	R	0.099 (0.329)	0.082 (0.500)	−0.148 (0.251)	−0.397 (0.020)
	L	−0.303 (0.085)	−0.405 (0.179)	−0.129 (0.278)	−0.345 (0.042)
Occipital	R	0.012 (0.478)	−0.143 (0.725)	−0.052 (0.410)	−0.353 (0.042)
	L	0.079 (0.362)	−0.417 (0.268)	−0.051 (0.409)	−0.408 (0.043)

*The levels of CBF volume were significantly correlated with the number of lobar CMBs at the left temporal lobe (r = −0.382, p = 0.046) in lvPPA, and at the right and the left temporal lobes (r = −0.345, p = 0.042; r = −0.368, p = 0.027, respectively), at the right and the left parietal lobes (r = −0.397, p = 0.020; r = −0.345, p = 0.042, respectively), and at the right and the left occipital areas (r = −0.353, p = 0.042; r = −0.408, p = 0.043, respectively) in LOAD*.

### Correlation Between the Number of Lobar CMBs and the Levels of CSF Markers in the Total AD and the Levels of NFL in the AD Subtypes

**Figure 3:**
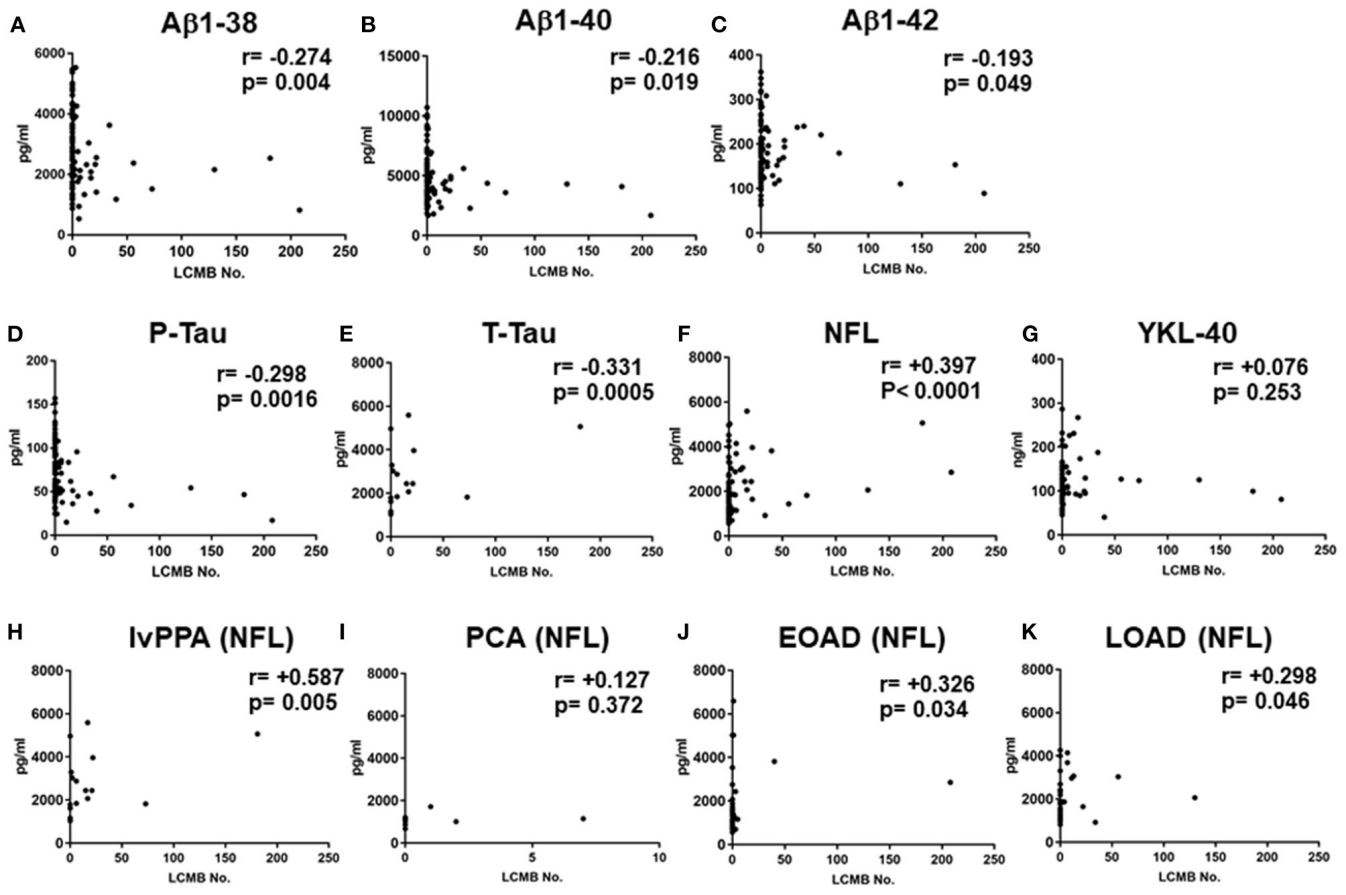
Correlations between the number of lobar cerebral microbleeds, and cerebrospinal fluid levels of Aβ1-38, Aβ1-40 Aβ1-42, P-Tau, T-Tau, NFL, and YKL-40 in the total AD subtypes, and CSF NFL in the lvPPA. Scatter plots presenting the correlations per patient between total number of lobar CMBs and CSF biomarkers, Aβ1-38 **(A)**, Aβ1-40 **(B)**, Aβ1-42 **(C)**, P-Tau **(D)**, T-Tau **(E)**, NFL **(F)**, and YKL-40 **(G)**. The number of CMBs and the levels of CSF NFL in the lvPPA **(H)**, the PCA **(I)** the EOAD **(J)**, and the LOAD **(K)**.

In the total AD subtypes, Spearman rank correlation tests revealed that the CSF levels of Aβ1-38, Aβ1-40, Aβ1-42, P-Tau and T-Tau were significantly negatively correlated with the amounts of lobar CMBs (*r* = −0.274, *p* = 0.004; *r* = −0.216, *p* = 0.019; *r* = −0.193, *p* = 0.049; *r* = −0.298, *p* = 0.0016; *r* = −0.331, *p* = 0.0005, respectively, by Spearman's rank correlation *t*-tests), while the CSF levels of NFL were significantly positively correlated with the number of lobar CMBs (*r* = +0.397, *p* < 0.0001: Spearman *t*-test), although YKL-40 showed no significant difference (Figures 3A–G). CSF levels of NFL in the lvPPA patients showed significantly positive correlation with the number of lobar CMBs (*r* = +0.587, *p* = 0.005), while the EOAD and LOAD also show significant differences (*r* = +0.326, *p* = 0.034; *r* = +0.298, *p* = 0.046) (Figures 3H–K).

## Discussion

The key findings of our study are as follows. First, the CSF levels of Aβ1-42 were significantly lower in combined the typical AD group and the atypical AD than in the ND, while the CSF levels of P-Tau, T-Tau, NFL, and YKL-40 were significantly higher in the typical AD and atypical AD groups compared to the ND group. Second, the CSF levels of NFL in the four AD subtypes were significantly higher than those of ND, and the CSF levels of NFL were significantly higher in lvPPA than in PCA, EOAD, and LOAD among the four AD subtypes. While the CSF levels of YKL-40 of the four AD subtypes were higher than ND, however, YKL-40 did not show significant differences among the four AD subtypes. Third, among the four clinical AD subtypes, lvPPA had the highest prevalence of lobar CMBs (50%), which were distributed across the frontal, temporal, parietal, and occipital lobe cortices with a left side predominance. Meanwhile, LOAD had a tendency of a higher number of lobar CMBs in the occipital lobe cortices bilaterally than the other AD subtypes, although did not show significant difference. Among the four AD subtypes, the total number of lobar CMBs in lvPPA and LOAD were significantly higher than those in PCA. Fourth, the CBF volumes in lvPPA were reduced in the frontal, temporal, and occipital lobe areas, compared to those in the other AD subtypes, with a significant left side predominance, while the CBF volumes of PCA were significantly lower in the bilateral occipital and parietal lobes areas than in those of LOAD. Fifth, we identified a significant inverse correlation between the number of lobar CMBs and the CBF volume in the left temporal area in lvPPA, and in the bilateral temporal, parietal, and occipital areas in LOAD; while those of PCA and EOAD showed similar inverse correlations, however, were not significant. The global ^11^C PiB retention had no significant difference among the four AD subtypes. We found that higher number of lobar CMBs in the AD patients were significantly associated with lower amount of the smaller peptides Aβ1-38 and higher amount of NFL, although Aβ1-40, Aβ1-42, P-Tau and T-Tau have been reported (51, 52). Furthermore, CSF levels of NFL in the lvPPA patients was higher in a significantly positive correlation with the number of lobar CMBs (*p* = 0.005), while the EOAD and LOAD also show significant differences (*p* = 0.034, *p* = 0.046, respectively).

We describe our considerations about the results in this study as follows. (1) We demonstrated that the CSF levels of NFL and YKL-40 were significantly higher in the four AD subtypes than in ND. Moreover, among the four AD subtypes, the CSF levels of NFL in lvPPA were significantly higher than those in the other AD subtypes. Additionally, CSF levels of NFL in the lvPPA patients were higher in significantly positive correlation with the number of lobar CMBs. (2) Lobar CMBs in the four AD subtypes were most frequently detected in lvPPA, followed by LOAD, PCA, and EOAD. The APOE ε4 allele had the highest frequency in EOAD, which had the lowest prevalence of lobar CMBs, suggesting that the prevalence of lobar CMBs may be related not only to APOE ε4 allele carrier status, but also to other factors, such as advanced aging and higher AAO of dementia (lvPPA and LOAD). (3) The lvPPA group showed a higher number of lobar CMBs in the frontal, temporal, and parietal lobe cortices, in contrast to those of EOAD and PCA, which may involve speech/language disturbances, resulting in logopenic speech, although the other AD subtypes did not show any laterality predominance of CMBs in distribution of cerebral cortices. Lobar microbleeds in typical amnestic LOAD and elderly people have been reported to be located predominantly in the occipital lobe cortices, presumably since aging and longevity might induce more severe CAA (5). (4) In lvPPA, AD pathology characterized by lobar CMBs with a clear left side predominance is likely to occur as a result of ischemic vascular pathology derived from lobar CMBs (53). However, although we observed high prevalence of lobar CMBs in left frontal areas, we observed a significant inverse correlation between CBF volume and the number of lobar CMBs in the left temporal region in lvPPA. In PCA, bilateral occipital and parietal lobes showed a decrease in CBF volumes, likely due to localized brain atrophy, possibly involving visuospatial cognitive syndromes. Consistent with three previously published reports (21–23), while the CSF levels of P-Tau, T-Tau, and NFL in all PCA patients were significantly higher than in the ND group, the lowest among all four AD groups was observed in PCA patients who also showed positive ^11^C PiB retention, suggesting that PCA, based upon Aβ pathology, may not be as aggressive a disease of tau pathology and axonal neurodegeneration as the other AD subtypes. For this reason, PCA of the amnestic AD type was considered to have unique differential pathology from that of typical AD even if compatible with AD criteria (54). The elevated CSF NFL levels were significantly related to longitudinal cognitive decline in AD and mild cognitive impairment (MCI) (25). Moreover, CSF NFL had a stronger correlation than those of T-Tau and P-Tau, leading to brain atrophy and progression of cognitive decline in AD (26). Pathologically, tau accumulation is reported to cause a great burden to cortical areas of the predominant hemisphere in lvPPA (55). With regards to lvPPA, the coexistence of AD pathology and argyrophilic thorny astrocytes clusters (ATAC) have been focused on intensely in tau immunoreactive pathology in fronto-temporo-parietal cortices as well as subcortical regions in lvPPA, suggesting that they may be markers of a process responsible for the prominent focal clinical manifestations of lvPPA based upon AD pathology (56, 57). Quite recently, Buciuc et al. reported that CAA pathology was the dominant risk factor of CMBs/SS (cortical superficial siderosis) in LPA (lvPPA) by neuroimaging-pathological analyses, they emphasized that CMB/SS were frequent in LPA patients (46%: 6/13) pathologically with moderate/severe CAA (58). They also described that they did not observe a higher frequency/number of CMBs/SS in the regions with the most severe CAA, nor observe a topolographic relationship between CMBs/SS location and regional PiB uptake or regional hypometabolism, although most CMBs/SS co-occurred with some degree of regional CAA. Their results were consistent with another neuroimaging-neuropathological study of CMBs/SS and CAA, where CMBs/SS occurred at the sites with reduced CAA (59). We found a significant inverse correlation between CBF volume and the number of lobar CMBs in the left temporal region in lvPPA, although most frequent prevalence of lobar CMBs was left frontal areas, which might imply that lobar CMBs were not necessarily determinant for decreased CBF volume, but regional brain atrophy might reflect the decrease of CBF volume.

We found that CSF levels of NFL were significantly higher in lvPPA than those of other AD subtypes after controlling for multiple comparison, additionally, CSF NFL elevated by the increased number of lobar CMBs in lvPPA patients, presumably that lobar CMBs and/or CAA pathology in lvPPA might involve secondary and/or indirectly neuronal and axonal degeneration based upon AD pathology. Indeed, in analyses of functional neuronal connectivity in lvPPA patients, not only the language network (posterior superior temporal gyrus and inferior frontal lobe) but also the working memory network (frontal regions, inferior parietal lobule, superior, and middle temporal gyri) have been shown to be widely disturbed with a left side predominance by a resting-state fMRI study (60). Our findings of lvPPA imply that left predominant hypoperfusion may occur due to left dominant brain atrophy causing by neuronal and axonal degeneration, and possibly and/or partly due to aberrant microcirculation causing by lobar CMBs and cerebrovascular injuries.

The limitation of this study was the absence of post-mortem autopsy analysis for any of the patients, which prevented us from performing pathological diagnosis to support our clinical diagnosis. Nonetheless, to ensure that the atypical AD group (lvPPA and PCA) did not include patients with CBS, PSP, FTD, DLB, or other neurodegenerative diseases that present with dementia, we performed a careful clinical diagnosis in strict accordance with the current global criteria (8, 9, 13–17).

## Data Availability Statement

All data generated or analyzed during this study are included in this published article.

## Ethics Statement

The studies involving human participants were reviewed and approved by the Gunma University Ethical Review Board for Medical Research Involving Human Subjects of Gunma University (Maebashi, Gunma, Japan), the Geriatrics Research Institute and Hospital (Maebashi, Gunma, Japan) and Maebashi Red Cross Hospital (Maebashi, Gunma, Japan). The patients/participants provided their written informed consent to participate in this study.

## Author Contributions

MI, HK, KN, YF, KM, KH, NF, MF, YH, SN, NH, YT, HT, YS, TY, YT, YA, MA, HY, KO, MT, and YI collected the clinical data, interpreted the data. MI wrote the manuscript. ET analyzed genomic DNA from the patient's blood samples and CSF biomarkers from the patient's CSF. ES and AK performed neuropsychological examinations. MI, SK, HK, TH, YT, and KI evaluated the neuroimaging information. KK, HS, and TS organized the neuroimaging systems. MI and YI performed the clinical data analysis and evaluated the specificities and neurological significances. All authors contributed to the article and approved the submitted version.

## Conflict of Interest

The authors declare that the research was conducted in the absence of any commercial or financial relationships that could be construed as a potential conflict of interest.

## Supplementary Material

Supplementary Table 1 Assay quality data of Inter-assay CVs (Coefficients of variation) %. Inter-assay CVs of Aβ1-42, Aβ1-40, and Aβ1-38 were <15, 15, and 20%, respectively. Inter-assay CVs of P-Tau, T-Tau, NFL, and YKL-40 were <15, 20, 20, and 15%, respectively.

Supplementary Table 2 MRI parameters. FA, Flip angle; ST, Slice thickness; TE, Time to echo; TI, Time for inversion; TR, Time to repeat.

Supplementary Table 3 Distribution of patients across field strengths and sequences. 1.5 and 3.0 T columns included all the patients scanned on these field strengths in the different AD subgroups. There was no significant difference among the four subgroups of AD across field strength and hemosiderin positive sequences.
