# Supplementary figures and images for: Cerebral Microbleeds, Cerebrospinal Fluid, and Neuroimaging Markers in Clinical Subtypes of Alzheimer's Disease
Source: Front Neurol. 2021 Apr 6;12:543866. doi: 10.3389/fneur.2021.543866 (PMC8056016; doi:10.3389/fneur.2021.543866)

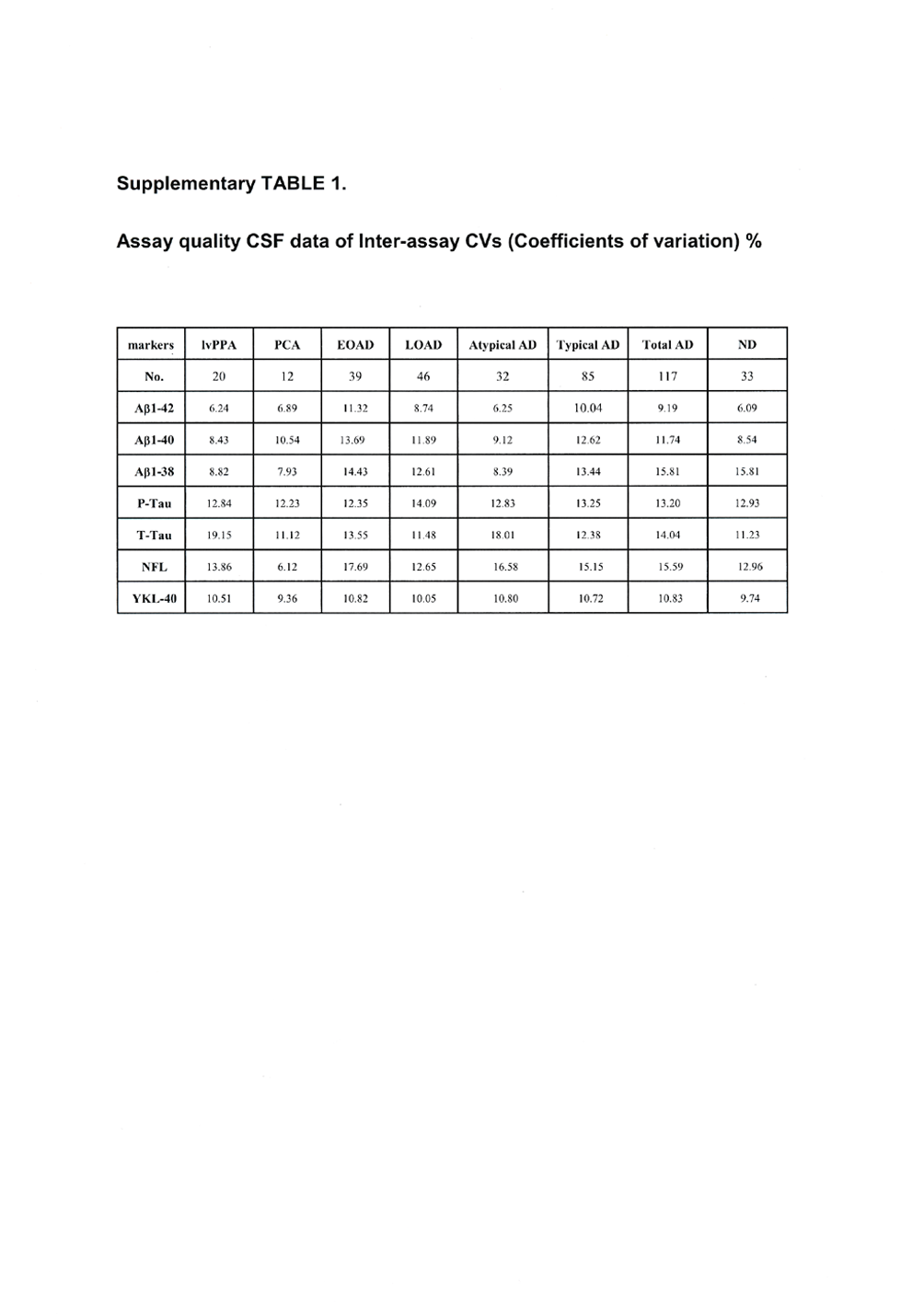

Supplement: Supplementary Table 1 — Assay quality data of Inter-assay CVs (Coefficients of variation) %. Inter-assay CVs of Aβ1-42, Aβ1-40, and Aβ1-38 were <15, 15, and 20%, respectively. Inter-assay CVs of P-Tau, T-Tau, NFL, and YKL-40 were <15, 20, 20, and 15%, respectively. [file Image_1.TIF]

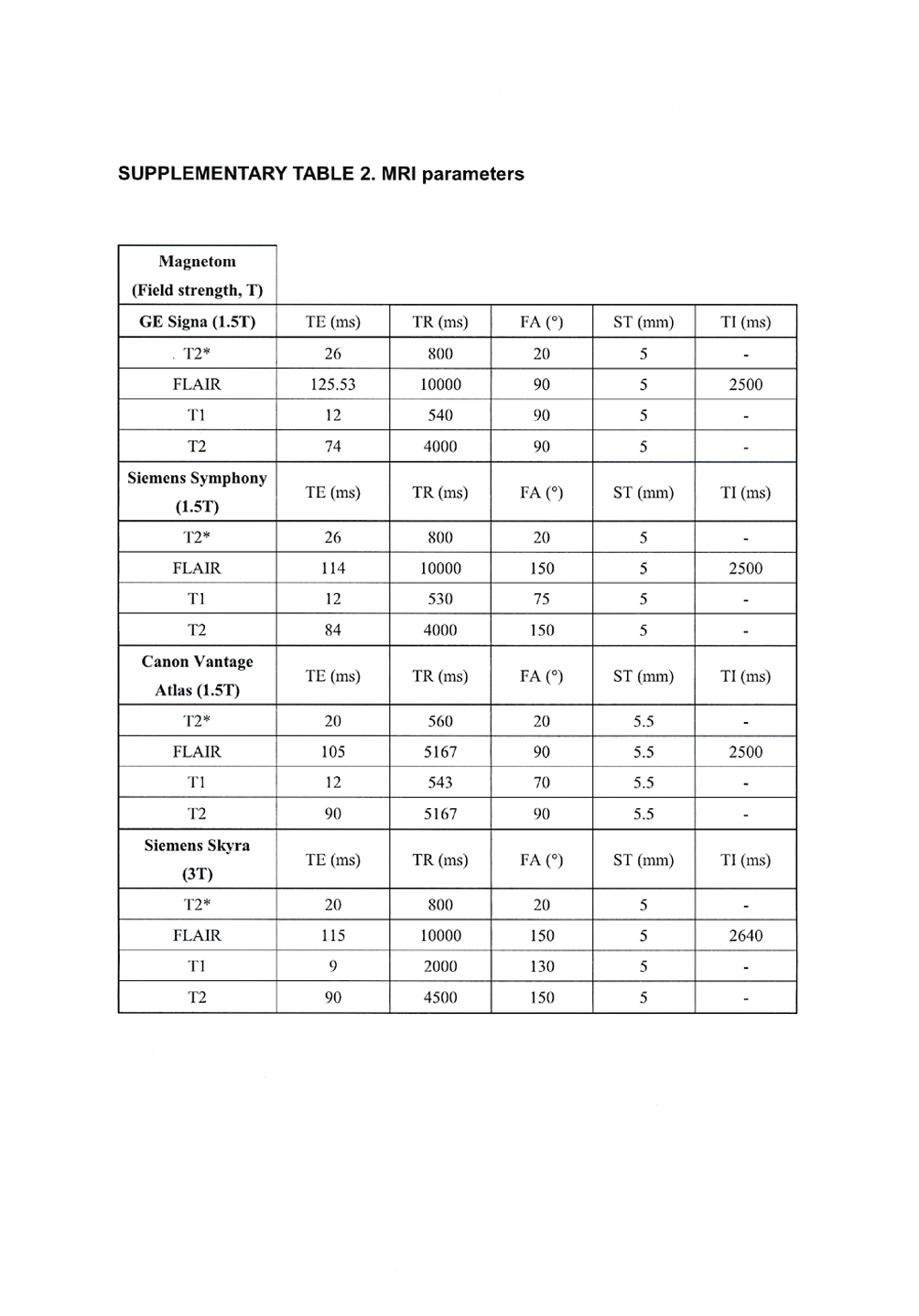

Supplement: Supplementary Table 2 — MRI parameters. FA, Flip angle; ST, Slice thickness; TE, Time to echo; TI, Time for inversion; TR, Time to repeat. [file Image_2.TIF]

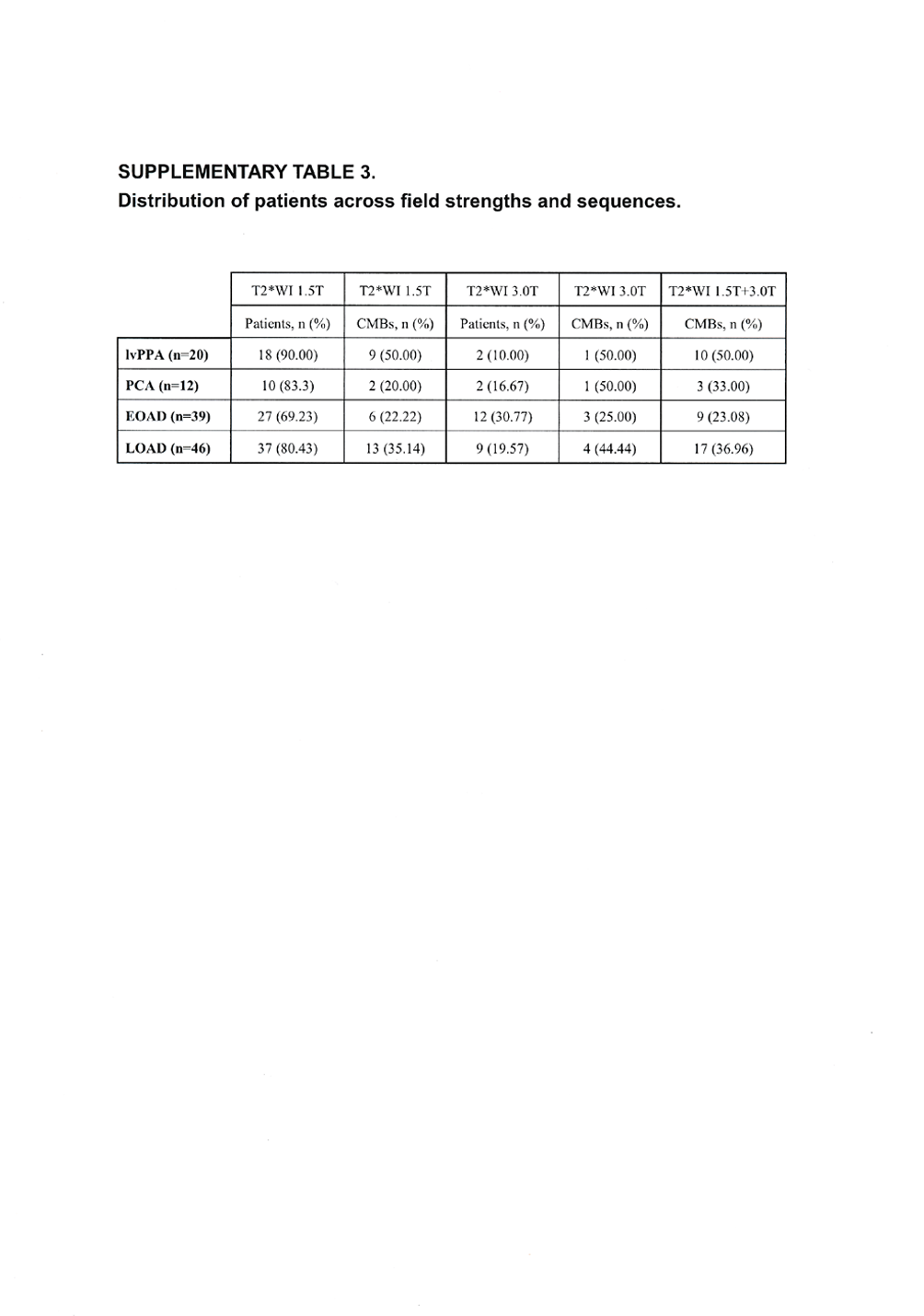

Supplement: Supplementary Table 3 — Distribution of patients across field strengths and sequences. 1.5 and 3.0 T columns included all the patients scanned on these field strengths in the different AD subgroups. There was no significant difference among the four subgroups of AD across field strength and hemosiderin positive sequences. [file Image_3.TIF]
